# Prognostic stratification of endometrial cancers with high microsatellite instability or no specific molecular profile

**DOI:** 10.3389/fonc.2023.1105504

**Published:** 2023-05-23

**Authors:** Jesus Gonzalez-Bosquet, S. John Weroha, Jamie N. Bakkum-Gamez, Amy L. Weaver, Michaela E. McGree, Sean C. Dowdy, Abimbola O. Famuyide, Benjamin R. Kipp, Kevin C. Halling, Siddhartha Yadav, Fergus J. Couch, Karl C. Podratz

**Affiliations:** ^1^Department of Obstetrics and Gynecology, University of Iowa, Iowa City, IA, United States; ^2^Division of Medical Oncology, Mayo Clinic, Rochester, MN, United States; ^3^Department of Obstetrics and Gynecology, Mayo Clinic, Rochester, MN, United States; ^4^Department of Quantitative Health Sciences, Mayo Clinic, Rochester, MN, United States; ^5^Department of Laboratory Medicine and Pathology, Mayo Clinic, Rochester, MN, United States

**Keywords:** *CCNA2*, ECPPF, *E2F1*, endometrial cancer, *FBXW7*, MSI, NSMP, *PPP2R1A*

## Abstract

**Objective:**

To identify high-risk disease in clinicopathologic low-risk endometrial cancer (EC) with high microsatellite instability (MSI-H) or no specific molecular profile (NSMP) and therapeutic insensitivity in clinicopathologic high-risk MSI-H/NSMP EC.

**Methods:**

We searched The Cancer Genome Atlas for DNA sequencing, RNA expression, and surveillance data regarding MSI-H/NSMP EC. We used a molecular classification system of *E2F1* and *CCNA2* expression and sequence variations in *POLE*, *PPP2R1A*, or *FBXW7* (ECPPF) to prognostically stratify MSI-H/NSMP ECs. Clinical outcomes were annotated after integrating ECPPF and sequence variations in homologous recombination (HR) genes.

**Results:**

Data were available for 239 patients with EC, which included 58 MSI-H and 89 NSMP cases. ECPPF effectively stratified MSI-H/NSMP EC into distinct molecular groups with prognostic implications: molecular low risk (MLR), with low *CCNA2* and *E2F1* expression, and molecular high risk (MHR), with high *CCNA2* and *E2F1* expression and/or *PPP2R1A* and/or *FBXW7* variants. The 3-year disease-free survival (DFS) rate was 43.8% in the MHR group with clinicopathologic low-risk indicators and 93.9% in the MLR group (*P*<.001). In the MHR group, wild-type HR genes were present in 28% of cases but in 81% of documented recurrences. The 3-year DFS rate in patients with MSI-H/NSMP EC with clinicopathologic high-risk indicators was significantly higher in the MLR (94.1%) and MHR/HR variant gene (88.9%) groups than in the MHR/HR wild-type gene group (50.3%, *P*<.001).

**Conclusion:**

ECPPF may resolve prognostic challenges for MSI-H/NSMP EC by identifying occult high-risk disease in EC with clinicopathologic low-risk indicators and therapeutic insensitivity in EC with clinicopathologic high-risk indicators.

## Introduction

Over the past 4 decades, prognostic stratification of endometrial cancer (EC) has progressively incorporated serous histopathologic findings, lymphatic metastases, lymphovascular space involvement, *TP53* variations, *ERBB2* expression, mismatch repair and homologous recombination (HR) repair deficiencies, and other variables (1–5). More recently, The Cancer Genome Atlas (TCGA) generated a molecular-based prognostic classification system (6). Despite the evolution of EC risk stratification, corresponding risk-adjusted therapy has minimally affected EC mortality rates. The American Cancer Society 2020 Annual Report concluded that “cancer survival has improved since the mid-1970s for all of the most common cancers except uterine cervix and uterine corpus” and that “stagnant survival rates reflect a lack of major treatment advances” (7). Addressing this lack of major treatment advances requires an enhanced understanding of pivotal tumor-specific molecular drivers to facilitate the discovery of target-specific therapeutics.

TCGA classification of EC identified 4 unique molecular subgroups: 1) *POLE* ultramutated, characterized by *POLE* variations; 2) microsatellite instability hypermutated, classified by mismatch repair deficiency and high microsatellite instability (MSI-H); 3) copy-number low, denoted by no specific molecular profile (NSMP); and 4) copy-number high, primarily typified by *TP53* variations and serouslike EC (6). Comparative prognoses associated with *POLE* ultramutated and copy-number high EC are contrasting, in which the prognosis is favorable for *POLE* ultramutated EC and substantially compromised for copy-number high EC. MSI-H and NSMP ECs have an intermediate prognosis but account for most EC tumors, thereby highly contributing to the annual EC mortality rate (6, 8–10). Therefore, determining the causes of treatment failures in MSI-H and NSMP ECs is paramount. Because MSI-H and NSMP tumors are predominantly low stage, with grade 1 and 2 histopathologic indicators (11), treatment failures may reflect our inability to identify clinicopathologic low-risk cases at high risk for recurrence (i.e., EC harboring occult high-risk disease). Radiotherapy (RT) and/or platinum-based chemotherapy (PbCT) are standard adjuvant treatment modalities for clinicopathologic high-risk MSI-H and NSMP EC. Treatment failures may also result from our inability to predict insensitivity to these standard adjuvant therapeutics (9, 12).

MSI-H EC generally harbors a high tumor mutation burden (TMB), and thus patients with MSI-H tumors are considered potential candidates for immunotherapy (6, 13–16). However, the high associated TMB presumably predicts a greater prevalence of HR gene variations, which portend sensitivity to DNA-damaging agents in cases with clinicopathologic high-risk indicators (17–20). Defining specific vulnerabilities in MSI-H tumors is necessary for decreasing the mortality rate in patients with this molecular profile. Furthermore, because of the absence of identifiable molecularly targetable pathways in NSMP, empiric therapy must be used for the largest EC cohort. Therefore, we used our previously described stratification system based on *E2F1* and *CCNA2* expression and sequence variations in *POLE*, *PPP2R1A*, and *FBXW7* (termed ECPPF) to molecularly stratify MSI-H and NSMP ECs (21). The primary aim of our study was to use ECPPF to identify MSI-H and NSMP EC cases with clinicopathologic low-risk indicators that are at risk for occult extrauterine disease and recurrence and those with clinicopathologic high-risk indicators that are insensitive to standard therapeutic regimens.

## Methods

### Study population

The initial report of the ECs curated in the TCGA database consisted of 239 cases with documented DNA sequencing, RNA expression, and sufficient surveillance data (6). According to TCGA molecular classification of these cases, 30 tumors had *POLE* variations, 58 had MSI-H, 62 had *TP53* variations (presumed copy-number high), and an estimated 89 had NSMP (i.e., copy-number low). We previously reported the integration of genomic, transcriptomic, and clinical outcomes data for these cases, which culminated in the generation of the ECPPF stratification system (21). However, the molecular-based stratification of clinical outcomes for these EC cases may have been biased by a relatively high percentage (26%) of tumors with high-risk *TP53* variations; therefore, we limited the current study population to only the 147 cases of MSI-H and NSMP EC, which consisted predominantly of early-stage, low-grade tumors. As previously reported, the EC tumors included in TCGA were surgically staged, and adjuvant therapy consisted of chemotherapy and/or RT, with 98% of patients who received chemotherapy receiving PbCT (6).

### Data acquisition

TCGA data relevant to EC (6) were downloaded as normalized, formatted, and organized data for integration and analysis as described in previous studies (21, 22). All data collection and processing, including the informed consent process, were performed after approval by the local institutional review board or ethics committee for each contributing institution and in accordance with TCGA Human Subjects Protection and Data Access Policies adopted by the National Cancer Institute and the National Human Genome Research Institute.

### Sequence variation analysis

Only validated sequence variations (or TCGA level 3 variations) were used for analysis (6). Sequence variant information was abstracted from exome-sequencing data generated with the Illumina Genome Analyzer GAIIx or HiSeq 2000 sequencing platforms (Illumina, Inc). Silent variations were excluded from the analysis, and only frame-shift insertions and deletions, in-frame insertions or deletions, missense, nonsense, nonstop, and splice-site variations were included in the study. For our analysis, the number of variations for each selected gene and for each patient were recorded.

### Gene expression

Normalized and log-transformed gene expression data were downloaded as level 3 RNA-sequencing data. These data were generated with Illumina HiSeq 2000 platforms and annotated with the hg19 version of the human genome. Statistical analyses of RNA expression data were performed with R software (The R Foundation) (23) and Bioconductor packages (Bioconductor) (24).

### Molecular stratification of MSI-H and NSMP EC

We previously reported the applicability of ECPPF for stratifying the unabridged EC cohort in TCGA independent of histomorphologic variables (21). Restricting the study population to MSI-H and NSMP EC rendered analysis of *POLE* variations expendable from the ECPPF profiling panel. The log-transformed gene expression equal to or greater than 2.75 for either or both *CCNA2* and *E2F1* was converted to the quantitative expression sum of *CCNA2*+*E2F1* and categorized as either low expression (sum<4.75) or high expression (sum≥4.75). Assessment of HR gene variations was restricted to the 10 most prevalent genes (*ATR*, *ATM*, *BRCA2*, *BRCA1*, *CDK12*, *BARD1*, *NBN*, *PALB2*, *CHEK1*, and *RAD51*) reportedly linked to EC and MSI-H (3, 25–28).

### Statistical analysis

Data were descriptively summarized as frequency and percentage for categorical variables and median (IQR) for continuous variables. Clinicopathologic and molecular risk factors were compared between the MSI-H and NSMP molecular classification groups by using χ^2^ or Fisher exact tests for categorical variables. Follow-up duration was calculated from the date of surgical resection to the date of first documented recurrence or latest follow-up. Disease-free survival (DFS) rates were estimated by using the Kaplan-Meier method and compared between groups with log-rank tests. Cox proportional hazards models were fit to evaluate the association between molecular parameters and the risk of recurrence; associations were summarized with hazard ratios and 95% CIs estimated from the models. All calculated *P* values less than .05 were considered statistically significant. The data were analyzed with SAS, v9.4 (SAS Institute Inc).

## Results

### Clinicopathologic stratification of MSI-H and NSMP EC

MSI-H (n=58) and NSMP (n=89) tumors had prevalent early-stage, low-grade histopathologic characteristics and 50% or less myometrial invasion (MI) (**Table 1**). The median (IQR) age of the 147 patients with these tumors was 60 (54-67) years. Recurrences were documented in 26 (18%) cases, and the median (IQR) time to recurrence after surgical resection was 13.9 (10.6-22.9) months. Among the 121 cases without evidence of recurrence, the median (IQR) follow-up period was 28.3 (15.7-44.8) months.

**Table 1:** Clinicopathologic and molecular characteristics of endometrial cancer with MSI-H and NSMP^a^.

Characteristic	Total (N=147)	MSI-H (n=58)	NSMP (n=89)	*P*
Age, y	60 (54-67)	60 (57-69)	60 (53-65)	.16
Stage				.23
I/II	121 (82.3)	45 (78)	76 (85)	
III/IV	26 (17.7)	13 (22)	13 (15)	
Grade				<.001
1	62 (42.2)	19 (33)	43 (48)	
2	58 (39.5)	19 (33)	39 (44)	
3	27 (18.4)	20 (35)	7 (8)	
MI				.99
≤50%	114 (77.6)	45 (78)	69 (78)	
>50%	33 (22.4)	13 (22)	20 (23)	
Clinicopathologic risk level^b^				.006
Low	91 (61.9)	28 (48)	63 (71)	
High	56 (38.1)	30 (52)	26 (29)	
Gene variations				
HR genes	33 (22.4)	26 (45)	7 (8)	<.001
* CTNNB1*	49 (33.3)	19 (33)	30 (34)	.91
* ARID1A*	55 (37.4)	22 (38)	33 (37)	.92
* KRAS*	30 (20.4)	14 (24)	16 (18)	.37
* PIK3CA*	82 (55.8)	42 (72)	40 (45)	.001
* PTEN*	101 (68.7)	37 (64)	64 (72)	.30
* TP53*	4 (2.7)	4 (7)	0 (0)	.02
ECPPF group				
Molecular low risk	90 (61.2)	27 (47)	63 (71)	.003
Molecular high risk^c^	57 (38.8)	31 (53)	26 (29)	
* CCNA2*/*E2F1*≥4.75	40 (27.2)	25 (43)	15 (17)	<.001
* PPP2R1A* variant	15 (10.2)	4 (7)	11 (12)	.28
* FBXW7* variant	9 (6.1)	6 (10)	3 (3)	.16
Surveillance time, mo				.01
To recurrence	13.9 (10.7-22.4)	11.7 (11.1-17.9)	17 (10.6-23.5)	
No recurrence	28.3 (15.7-44.8)	28.5 (15.5-61.6)	28.3 (16.2-40.8)	

ECPPF, stratification according to E2F1 and CCNA2 expression and sequence variations in POLE, PPP2R1A, and FBXW7; HR, homologous recombination; MI, myometrial invasion; MSI-H, high microsatellite instability; NSMP, no specific molecular profile.

a Age and surveillance time are summarized as median (IQR). All other values are No. (%), and comparisons were made with χ^2^ or Fisher exact tests.

b Clinicopathologic low risk: stage I, grade 1/2, <75% MI. Clinicopathologic high risk: stage I, grade 1/2, ≥75% MI; stage I, grade 3; and stage II-IV.

c Combinations of CCNA2/E2F1≥4.75 and FBWX7 and/or PPP2R1A variant subgroups were present in the MSI-H (n=4 combinations) and NSMP (n=3 combinations) endometrial cancer tumors.

**Figure 1:**
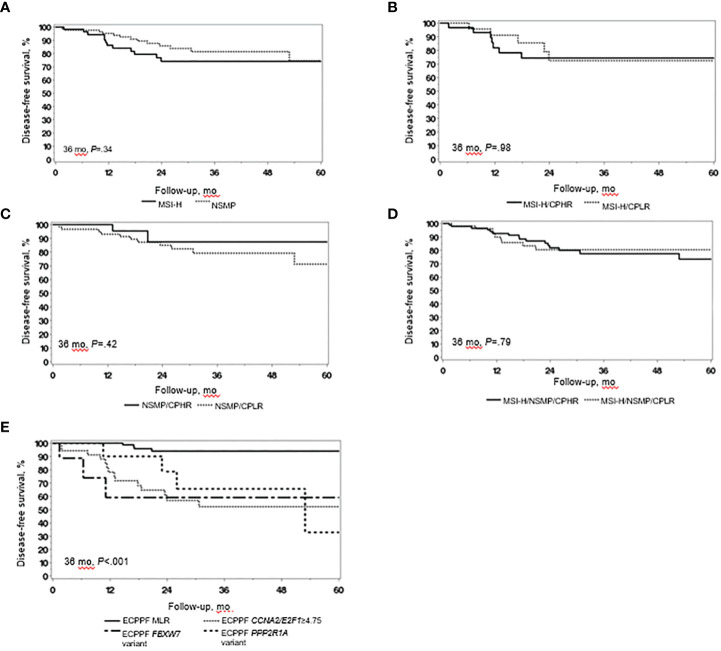
Clinical outcomes in endometrial cancer (EC) with high microsatellite instability (MSI-H) and no specific molecular profile (NSMP). **(A)** Disease-free survival (DFS) of patients with MSI-H EC (n=58) and NSMP EC (n=89). **(B)** DFS of patients with MSI-H EC with clinicopathologic low-risk (CPLR) indicators (n=28) and clinicopathologic high-risk (CPHR) indicators (n=30). **(C)** DFS of patients with NSMP EC with CPLR (n=63) and CPHR (n=26) indicators. **(D)** DFS of patients with combined MSI-H/NSMP EC with CPLR (n=91) and CPHR (n=56) indicators. **(E)** DFS of patients with combined MSI-H/NSMP EC (N=147) stratified by ECPPF into the molecular low-risk (MLR) group, characterized by *CCNA2*/*E2F1*<4.75 (n=90), and the following molecular high-risk subgroups: *CCNA2*/*E2F1*≥4.75 (n=34), *FBXW7* variant (n=14), and *PPP2R1A* variant (n=9). Kaplan-Meier curves for DFS were compared with log-rank tests.

We integrated tumor stage, grade, and MI data from the MSI-H and NSMP cases to analyze tumors with *clinicopathologic low-risk indicators* (i.e., stage I, grade 1/2, and <75% MI) and *clinicopathologic high-risk indicators* (i.e., stage I, grade 1/2, and ≥75% MI; stage I, grade 3; or stage II-IV). Although grade 3 histopathologic and clinicopathologic high-risk indicators were more prevalent in MSI-H tumors than in NSMP tumors, the 3-year DFS rates for patients with MSI-H and NSMP EC did not significantly differ (*P*=.34) (**Figure 1A**). The 3-year DFS rate (95% CI) was similar for patients with MSI-H tumors with clinicopathologic low-risk indicators and those with high-risk indicators (72.3% [53.9%-97.0%] vs 74.2% [59.4%-92.7%], *P*=.98) (**Figure 1B**). The 3-year DFS rate (95% CI) also did not significantly differ between patients with NSMP tumors with clinicopathologic low-risk indicators and those with high-risk indicators (79.0% [67.9%-92.0%] vs 87.3% [71.8%-100.0%], *P*=.42) (**Figure 1C**). Likewise, the 3-year DFS rate for the combined cohort of patients with MSI-H and NSMP ECs did not differ between those with clinicopathologic low-risk indicators (n=91) and those with high-risk indicators (n=56, *P*=.79) (**Figure 1D**).

### ECPPF stratification of MSI-H/NSMP EC

ECPPF stratified MSI-H/NSMP cases (N=147) into 2 distinct molecular groups with prognostic implications: 1) molecular low risk (MLR), characterized by low *CCNA2* and/or *E2F1* expression (i.e., *CCNA2*+*E2F1* quantitative expression sum<4.75), and 2) molecular high risk (MHR). The MHR group was further stratified into 3 subgroups: 1) *CCNA2*/*E2F1*≥4.75, characterized by high *CCNA2* and/or *E2F1* expression (i.e., *CCNA2*+*E2F1* quantitative expression sum≥4.75); 2) *PPP2R1A* variant; and 3) *FBXW7* variant. The 3-year DFS rate of the MLR group (n=90) was significantly higher than that of the MHR subgroups (*P*<.001) (**Figure 1E**). The hazard ratio (95% CI) was 10.48 (3.46-31.77) for the *CCNA2*/*E2F1*≥4.75 subgroup, 16.59 (3.67-74.99) for the *FBWX7* variant subgroup, and 7.74 (1.93-30.95) for the *PPP2R1A* variant subgroup (reference: MLR subgroup, *P*<.001).

### ECPPF stratification of MSI-H EC

**Figure 2:**
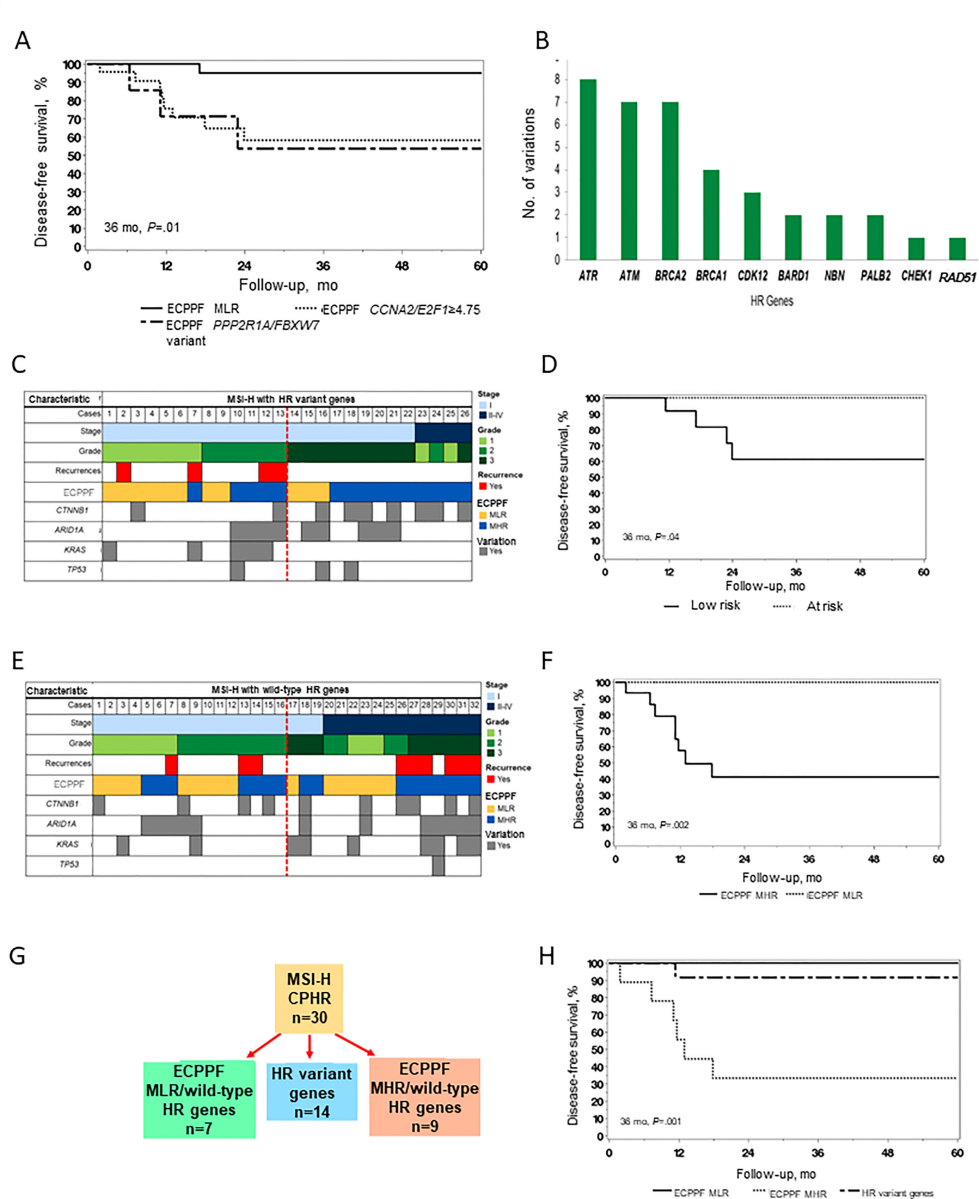
Molecular stratification and clinical outcomes of endometrial cancer (EC) with high microsatellite instability (MSI-H). **(A)** Disease-free survival (DFS) of patients with MSI-H EC (n=58) according to the ECPPF-stratified molecular low-risk (MLR) group, characterized by *CCNA2*/*E2F1*<4.75 (n=27), and the following molecular high-risk (MHR) subgroups: *CCNA2*/*E2F1*≥4.75 (n=22) and combined *PPP2R1A*/*FBXW7* variant (n=9). **(B)** Number of documented sequence variations in select homologous recombination (HR) genes in the MSI-H cohort. **(C)** Pattern of stage, grade, recurrences, ECPPF-stratified MLR and MHR groups, and variations in *CTNNB1*, *ARID1A*, *KRAS*, and *TP53* genes in cases of MSI-H EC with HR variant genes (red line delineates between stage I, grade 1/2 and other tumors). **(D)** DFS of patients with MSI-H EC with HR variant genes according to low-risk (n=13) and at-risk (n=13) tumors. **(E)** Pattern of stage, grade, recurrences, ECPPF-stratified MLR and MHR groups, and variations in *CTNNB1*, *ARID1A*, *KRAS*, and *TP53* genes in cases of MSI-H EC with wild-type HR genes (red line delineates between stage I, grade 1/2 and other tumors). **(F)** DFS of patients with MSI-H EC with wild-type HR genes according to ECPPF-stratified MLR (n=16) and MHR (n=16) groups. **(G)** ECPPF stratification of MSI-H EC cases with clinicopathologic high-risk indicators (CPHR) according to MLR and MHR groups and HR variant gene status. **(H)** DFS of patients with MSI-H EC with clinicopathologic high-risk indicators according to ECPPF-stratified MLR group with wild-type HR genes (n=7), MLR group with HR variant genes (n=14), and MHR group with wild-type HR genes (n=9). Kaplan-Meier curves for DFS were compared with log-rank tests.

We assessed clinical outcomes according to the ECPPF-stratified groups for MSI-H cases (**Figure 2A**). The 3-year DFS rate (95% CI) was 95.0% (85.9%-100.0%) for the MLR group, 58.2% (39.4%-85.8%) for the *CCNA2*/*E2F1*≥4.75 subgroup, and 53.6% (25.7%-100.0%) for the combined *PPP2R1A*/*FBXW7* variant subgroups (*P*=.01).

### Integrating ECPPF and HR gene variations in MSI-H EC

Among 58 MSI-H cases, 26 (45%) had at least 1 HR gene variation (**Table 1**). *ATR*, *ATM*, *BRCA2*, and *BRCA1* had the most sequence variations (**Figure 2B**). We used ECPPF to compare clinical outcomes of patients with stage I, grade 1/2 (i.e., *low-risk*) MSI-H EC tumors with HR variant genes and those with stage I, grade 3 or stage II-IV (i.e., *at-risk*) MSI-H EC tumors with HR variant genes (generally adjuvant therapy candidates) (**Figure 2C**). Most of the at-risk MSI-H cases with HR variant genes were stratified by ECPPF as MHR; however, no recurrences were documented in these cases. In contrast, several recurrences were documented in the low-risk MSI-H cases with HR variant genes, and most of these were stratified by ECPPF as MHR (**Figure 2C**). The 3-year DFS rate was also significantly lower for patients with low-risk MSI-H tumors with HR variant genes than those with at-risk tumors (*P*=.04) (**Figure 2D**).

MSI-H tumors with wild-type HR genes were equally distributed among low-risk and at-risk cases. ECPPF stratification of these cases showed differential outcomes for the MLR and MHR groups (**Figure 2E**). No recurrences were documented in the MSI-H cases with wild-type HR genes that were ECPPF-stratified as MLR, but recurrences were documented in most of the ECPPF-stratified MHR cases. The 3-year DFS rate (95% CI) for patients with MSI-H tumors with wild-type HR genes that were ECPPF-stratified as MHR was 41.0% (21.4%-78.6%), which was significantly lower than that for patients with ECPPF-stratified MLR EC (*P*=.002) (**Figure 2F**).

Clinical outcomes were assessed after segregating MSI-H tumors with clinicopathologic high-risk indicators and HR variant genes (n=30). Generally, patients with these tumors are candidates for adjuvant therapy. Clinicopathologic high-risk MSI-H cases were ECPPF-stratified as MLR, HR variant gene, and MHR groups (**Figure 2G**). The 3-year DFS rate (95% CI) was 100% (100.0%-100.0%) for the MLR group, 91.7% (77.3%-100.0%) for the HR variant gene group, and 33.3% (13.2%-84.0%) for the MHR group (*P*=.001) (**Figure 2H**).

### ECPPF stratification of NSMP EC

**Figure 3:**
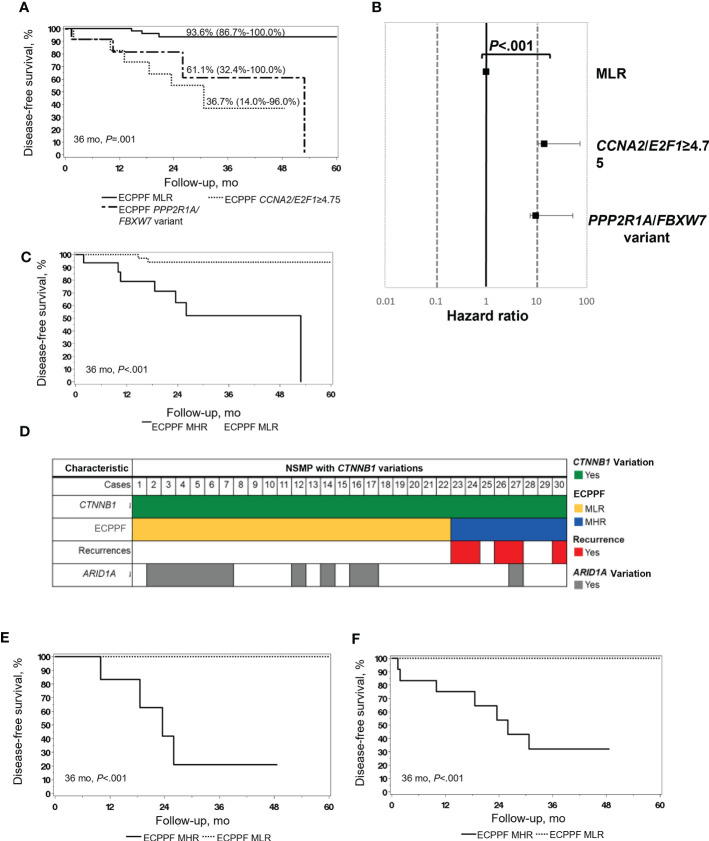
Molecular stratification and clinical outcomes of endometrial cancer (EC) with no specific molecular profile (NSMP). **(A)** Disease-free survival (DFS) of patients with NSMP EC (n=89) according to the ECPPF-stratified molecular low-risk (MLR) group, characterized by *CCNA2*/*E2F1*<4.75 (n=63), and the following molecular high-risk (MHR) subgroups: *CCNA2*/*E2F1*≥4.75 (n=12) and combined *PPP2R1A*/*FBXW7* variant (n=14). DFS (95% CI) at 36 months is indicated for each group. **(B)** Forest plot of Cox proportional hazard ratios for the 3 ECPPF-stratified groups (reference: *CCNA2*/*E2F1*<4.75). **(C)** DFS of patients with stage I, grade 1/2, ≤50% myometrial invasion (MI) NSMP EC (n=55) according to ECPPF-stratified MLR (n=39) and MHR (n=16) groups. **(D)** Pattern of recurrences, ECPPF-stratified MLR and MHR groups, and variations in *CTNNB1* and *ARID1A* in cases of NSMP with *CTNNB1* variations (n=30). **(E)** DFS of patients with stage I, grade 1/2, ≤50% MI NSMP EC with *CTNNB1* variations (n=21) according to ECPPF-stratified MLR (n=15) and MHR (n=6) groups. **(F)** DFS of patients with NSMP EC with *CTNNB1* and *ARID1A* variations (n=52) according to ECPPF-stratified MLR (n=39) and MHR (n=13) groups. Kaplan-Meier curves for DFS were compared with log-rank tests.

Clinicopathologic low-risk indicators were present more often in NSMP (n=63, 71%) than in MSI-H (n=28, 48%) tumors (*P*=.006) (**Table 1**) but failed to predict superior DFS (**Figure 1C**). ECPPF stratification into the MLR group and MHR *CCNA2*/*E2F1*≥4.75 and combined *PPP2R1*/*FBXW7* variant subgroups resulted in significantly different 3-year DFS rates for patients with NSMP EC (*P*<.001) (**Figure 3A**). The 3-year DFS rate (95% CI) was 93.6% (86.7%-100.0%) for the MLR group, 36.7% (14.0%-96.0%) for the *CCNA2*/*E2F1*≥4.75 subgroup, and 61.1% (32.4%-100.0%) for the combined *PPP2R1A*/*FBXW7* variant subgroups. Furthermore, the hazard ratio (95% CI) was 14.29 (3.39-60.19) for the *CCNA2*/*E2F1*≥4.75 subgroup and 9.72 (2.16-43.74) for the combined *PPP2R1A*/*FBXW7* variant subgroups (reference: MLR subgroup, *P*<.001) (**Figure 3B**).

### ECPPF stratification of early-stage, low-grade NSMP EC

Because 55 of 89 (62%) NSMP cases were stage I, grade 1/2 with 50% or less MI, which generally confers excellent outcomes without adjuvant chemotherapeutic treatment, we next used ECPPF to identify cases of occult high-risk disease in conventionally low-risk EC. ECPPF stratification of these NSMP early-stage, low-grade cases into MLR and MHR groups resulted in significantly different 3-year DFS rates (*P*<.001) (**Figure 3C**). The 3-year DFS rate (95% CI) was 93.9% (86.1%-100.0%) for the MLR group and 51.8% (29.7%-90.5%) for the MHR group. The hazard ratio (95% CI) for the MHR group was 12.10 (2.46-59.42) (reference: MLR group, *P*=.002).

### ECPPF stratification of CTNNB1 variations in NSMP EC

Among 30 *CTNNB1*-variant NSMP cases, all were grade 1/2, and 22 were ECPPF-stratified as MLR (15 stage I with ≤50% MI, 4 with >50% MI, and 3 stage II/III), and 8 were ECPPF-stratified as MHR (all stage I, and 2 with >50% MI). All documented *CTNNB1*-variant–associated recurrences occurred in the MHR group (**Figure 3D**). The 3-year DFS rate for patients with early-stage, low-grade NSMP tumors with *CTNNB1* variations significantly differed between the MLR and MHR groups (*P*<.001) (**Figure 3E**). ECPPF stratification of NSMP tumors with *ARID1A* and/or *CTNNB1* variations into MLR (n=39) and MHR (n=13) groups showed that all documented recurrences occurred in the MHR group. Consequently, the 3-year DFS rate (95% CI) for the MHR group was 32.1% (12.9%-80.3%), which was significantly lower than that for the MLR group (*P*<.001) (**Figure 3F**).

### ECPPF stratification predicts recurrence in MSI-H/NSMP EC with clinicopathologic low-risk indicators

**Figure 4:**
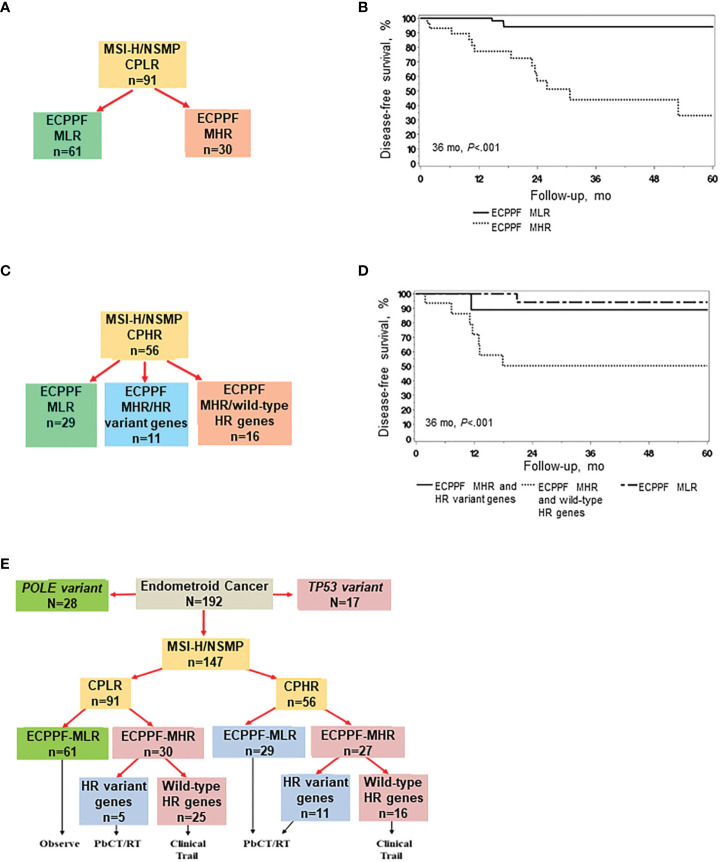
ECPPF stratification of endometrial cancer (EC) with high microsatellite instability (MSI-H) or no specific molecular profile (NSMP) and clinicopathologic low-risk (CPLR) or high-risk (CPHR) indicators. CPLR indicators comprise stage I, grade 1/2, <75% myometrial invasion (MI) tumors, and CPHR indicators comprise stage I, grade 1/2, ≥75% MI; stage I, grade 3; and stage II-IV tumors. **(A)** Stratification of MSI-H/NSMP cases with CPLR indicators according to the ECPPF-stratified molecular low-risk (MLR) and molecular high-risk (MHR) groups. **(B)** Disease-free survival (DFS) of patients with MSI-H/NSMP EC with CPLR indicators according to the ECPPF-stratified MLR (n=61) and MHR (n=30) groups. **(C)** Stratification of MSI-H/NSMP EC cases with CPHR indicators according to the ECPPF-stratified MLR group, MHR group with homologous recombination (HR) variant genes, and MHR group with wild-type HR genes. **(D)** DFS of patients with MSI-H/NSMP EC with CPHR indicators according to the ECPPF-stratified MLR group (n=29), MHR group with HR variant genes (n=11), and MHR group with wild-type HR genes (n=16). **(E)** ECPPF and HR variant gene stratification of MSI-H/NSMP cases with CPLR and CPHR indicators. Linkage to molecular-based treatment options is shown for each stratified group. Kaplan-Meier curves for DFS were compared with log-rank tests. PbCT indicates platinum-based chemotherapy; RT, radiotherapy.

ECPPF stratification of MSI-H/NSMP EC with clinicopathologic low-risk indicators (n=91) into the MLR and MHR groups (**Figure 4A**) yielded distinctly diverse clinical outcomes. The 3-year DFS rate (95% CI) was 93.9% (87.4%-100.0%) for the MLR group (n=61) and 43.8% (26.1%-73.6%) for the MHR group (n=30) (*P*<.001, **Figure 4B**). The hazard ratio (95% CI) for the MHR group was 12.37 (3.54-43.30) (reference: MLR group, *P*<.001).

### ECPPF stratification and HR variant gene status predict treatment sensitivity in MSI-H/NSMP EC with clinicopathologic high-risk indicators

We next assessed treatment sensitivity according to ECPPF stratification and HR variant gene status (**Figure 4C**). The 3-year DFS rate (95% CI) was significantly higher for the ECPPF-stratified MLR group (94.1% [83.6%-100.0%]) and the MHR group with HR variant genes (88.9% [70.6%-100.0%]) than that for the ECPPF-stratified MHR group with wild-type HR genes (50.3% [29.8%-84.6%]; *P*<.001) (**Figure 4D**).

### ECPPF stratification, clinicopathologic indicators, and wild-type HR gene status predict treatment sensitivity in MSI-H/NSMP EC

Segregating MSI-H and NSMP cases according to clinicopathologic low-risk and high-risk indicators first and then according to ECPPF-stratified MLR, MHR with HR variant gene, and MHR with wild-type HR gene groups may provide a molecular-based strategy for managing EC. Of 26 total documented recurrences, 21 (81%) occurred in the 41 (51%) ECPPF-stratified MHR group tumors with wild-type HR genes, including 14 of 25 (56%) of these tumors with clinicopathologic low-risk indicators.

Integrating ECPPF molecular stratification and treatment algorithms for MSI-H/NSMP EC in a hypothetical but similar population would most likely entail the following molecular-based therapy recommendations: observation for 41% (67% with clinicopathologic low-risk indicators) of patients, RT and/or PbCT for 31% of patients (including 5% with clinicopathologic low-risk indicators), and innovative clinical trials for 28% of patients (including 27% with clinicopathologic low-risk indicators) (**Figure 4E**).

## Discussion

Endometrioid histopathologic characteristics are present in more than 80% of EC tumors, with most classified as MSI-H or NSMP, low stage, and low grade (6, 11, 29). The observation that MSI-H/NSMP EC with clinicopathologic low-risk indicators accounted for most of the MSI-H/NSMP recurrences denotes major limitations in conventional risk assignment strategies. Improving clinical outcomes requires the identification of molecular vulnerabilities to permit target-specific risk designation and choice of therapeutics. Consequently, we assessed our previously described ECPPF molecular stratification system for its ability to resolve MSI-H/NSMP EC prognoses (21).

Because sensitivity to RT and chemotherapeutics are in part predicated on attenuated DNA-damage repair pathway elements (18, 30, 31), we integrated HR variant gene status into our stratification system. Our findings show that ECPPF prognostically stratifies MSI-H and NSMP ECs and that integration of HR variant gene status may facilitate development of new molecular-based therapeutic strategies. The ECPPF-stratified MHR group identified cases at high risk for recurrence in those conventionally classified as clinicopathologic low risk. ECPPF stratification also identified NSMP EC with variant *CTNNB1* as a subgroup with a high risk of recurrence and the MHR group with wild-type HR genes as insensitive to standard adjuvant PbCT and/or RT. In contrast, the ECPPF-stratified MLR group predicted sensitivity to standard therapies (PbCT and/or RT) in EC with clinicopathologic high-risk indicators and/or HR variant genes.

The high TMB associated with MSI-H EC has understandably led to embracing immunotherapy in persistent or recurrent disease (13–16). High TMB also predicts an increased prevalence of HR variant genes and most likely sensitivity to RT and DNA-damaging agents (17). Consistent with this precept, recent studies reported improved clinical outcomes with RT in patients with MSI-H EC (32, 33). In the current study, ECPPF effectively stratified MSI-H cases, in which patients in the MHR group with early-stage, low-grade tumors, who are rarely candidates for adjuvant therapy, had a poor DFS rate. Moreover, the ECPPF-stratified MLR and MHR groups with HR variant genes and clinicopathologic high-risk indicators had higher DFS rates than did the MHR group with wild-type HR genes and clinicopathologic high-risk indicators.

These observations advocate for strategizing treatment options for MSI-H and NSMP EC tumors with clinicopathologic low-risk indicators according to ECPPF-stratified MLR and MHR groups. Thus, pending HR gene status, conservative surveillance is recommended for the MLR group, and early therapeutic intervention (innovative clinical trials) is recommended for the MHR group. Furthermore, a therapeutic strategy for MSI-H and NSMP EC with clinicopathologic high-risk indicators would consist of treatment with contemporary PbCT and/or RT for patients with ECPPF-stratified MLR tumors and MHR tumors with HR variant genes but innovative clinical trials for those with ECPPF-stratified MHR tumors with wild-type HR genes.

Although NSMP EC is primarily early stage and low grade, it is associated with an intermediate prognosis (6, 8–10). This paradox suggests that its high recurrence rate is linked to unrecognized high-risk disease in conventionally classified low-risk EC. ECPPF appeared to effectively stratify NSMP EC cases, particularly for early-stage, low-risk tumors. The MLR group had a distinctly favorable DFS rate over that of the MHR group with occult high-risk disease. *CTNNB1* variations in low-grade NSMP EC are reportedly associated with poor patient outcomes (18, 34, 35). ECPPF efficiently stratified NSMP tumors with variant *CTNNB1*, and all documented recurrences occurred in the MHR group. ECPPF appears to provide a molecular profile for TCGA-designated copy-number low tumors by identifying occult high-risk disease within conventional low-risk cases and high-risk disease that is insensitive to standard adjuvant PbCT and/or RT.

The recently published version of the National Comprehensive Cancer Network guidelines encourages molecular assessment of EC and states that probing for *POLE* hotspot variations and performing immunohistochemical analysis of mismatch repair deficiency and wild-type *TP53* expression may identify MSI-H and NSMP, respectively (36). However, without specific profiling of adverse molecular drivers, targetable vulnerabilities remain unrecognized. Therefore, ECPPF classification provides new profiling insights to refine prognoses and improve therapeutic efficacy.

The predominant challenges for treating MSI-H and NSMP ECs are distinguishing occult high-risk disease in conventionally characterized histomorphologic low-risk EC and predicting insensitivity of EC with clinicopathologic high-risk indicators to contemporary therapies (12). The 43.8% 3-year DFS rate of the ECPPF-stratified MHR group with clinicopathologic low-risk indicators is the first evidence that molecular stratification can distinguish high-risk disease in conventionally classified low-risk MSI-H/NSMP EC. Importantly, 81% of documented recurrences occurred in the ECPPF-stratified MHR group with wild-type HR genes which accounted for only 28% of all TCGA MSI-H/NSMP EC cases. Furthermore, our analysis of MSI-H/NSMP EC with clinicopathologic high-risk indicators showed that the 3-year DFS rate of the MLR group and MHR group with HR variant genes was considerably disparate from that of the MHR group with wild-type HR genes. This finding is consistent with treatment sensitivity to standard therapeutic regimens in the MLR group and MHR group with HR variant genes and treatment insensitivity in the MHR group with wild-type HR genes. These observations introduce a potential molecular-based paradigm shift for precision therapeutics in MSI-H and NSMP ECs. On the basis of our previously reported molecular schematic of disrupted EC signaling pathways (21), reasonable therapeutic options may include modulators of the CCNA2-E2F1-CIP2A axis or PP2A activity and FBXW7 substrates alone or in combination with PbCT or PARP inhibitors (27, 36–40).

Strengths of our analyses include the size of the patient population, centralized pathology review, and availability of robust genomic and transcriptomic databases. Assessing the primary clinical outcome of DFS rather than overall survival is an additional strength of the study that afforded analysis of limitations in contemporary staging, prognostication, and therapeutic efficacy. Limitations of this study included the variability and length of follow-up periods. Because the upper quartile of time to recurrence exceeded the lower quartile of follow-up time, the extent of recurrences in our study population may be underestimated. Recent meta-analyses of prognostic indicators independent of the TCGA signature suggest that lymphovascular space involvement (LVSI) has prognostic value for EC and that deep MI may be a predictor of recurrence (41, 42). LVSI information was not accessible in the TCGA study population and warrants inclusion in subsequent ECPPF stratification studies. In previously reported multivariate analyses incorporating the ECPPF elements with numerous traditional risk factors, deep MI was not an independently significant risk factor (21, 43). Moreover, definitive information in TCGA regarding treatment strategies for the patients was not available. However, because specimens were submitted chiefly from comprehensive cancer centers, it is reasonable to assume that the therapies were consistent with contemporary established guidelines. Such therapies include surgical staging and adjuvant RT and/or chemotherapy in patients with clinically identified at-risk disease; as such, 98% of patients receiving chemotherapy received PbCT (6). Knowledge of the sites of recurrence (e.g., local, regional, or distant) would have also been preferable for this study, particularly for tumors with predominantly clinicopathologic low-risk indicators.

In summary, ECPPF identifies occult high-risk disease in MSI-H/NSMP EC with clinicopathologic low-risk indicators and appears to efficiently triage treatment sensitivity of MSI-H/NSMP EC with clinicopathologic high-risk indicators to contemporary adjuvant therapies. Consequently, ECPPF stratification confers a new therapeutic paradigm for MSI-H/NSMP EC: 1) observation only for MLR cases with clinicopathologic low-risk indicators; 2) PbCT and/or RT for MHR cases with HR variant genes and clinicopathologic low-risk indicators, MLR cases with clinicopathologic high-risk indicators, and MHR cases with HR variant genes; and 3) innovative clinical trials for MHR cases with wild-type HR genes and clinicopathologic low-risk or high-risk indicators.

## Expanded gene symbols

*CCNA2*, cyclin A2; *CTNNB1*, catenin beta 1; *E2F1*, E2F transcription factor 1; *FBXW7*, F-box and WD repeat domain-containing protein 7; *POLE*, DNA polymerase epsilon, catalytic subunit; *PPP2R1A*, protein phosphatase 2 scaffold subunit alpha.

## Data availability statement

The datasets presented in this study can be found in online repositories. The names of the repository/repositories and accession number(s) can be found in the article.

## Author contributions

Conceptualization: JG-B and KP; Data curation: JG-B and KP; Formal analysis: JG-B, JB-G, BK, KH, and KP; Funding acquisition: SD, AF, and KP; Investigation: JG-B, SW, JB-G, AW, MM, SD, AF, BK, KH, SY, FC, and KP; Methodology: JG-B, BK, KH, and KP; Project administration: KP; Resources: JG-B and KP; Software: JG-B, AW, and MM; Supervision: KP; Pathology: BK, KH, and FC; Writing – original draft: JG-B, JB-G, AW, MM, BK, KH, SY, and KP; Writing – review and editing: JG-B, SW, JB-G, AW, MM, SD, AF, BK, KH, SY, FC, and KP. All authors contributed to the article and approved the submitted version.
